# Navigating autonomy, privacy, and ageism in robot home care with aged users: A preliminary analysis of ROB‐IN

**DOI:** 10.1111/bioe.13340

**Published:** 2024-07-31

**Authors:** Belen Liedo

**Affiliations:** ^1^ Institute of Philosophy Spanish National Research Council Madrid Madrid Spain

**Keywords:** ageism, assistive robots, ethics of care, privacy: relational autonomy, roboethics

## Abstract

In this article, I propose an ethical analysis of assistive domestic robots for older users. In doing so, I illustrate my inquiry with the example of ROB‐IN assistive robot. ROB‐IN is a Spanish project which is devoted to developing a robot that will perform in the private home of nondependent, aged users. It is aimed to help people in their daily activities and contribute to appropriate health monitoring. One of their potentially most useful features is related to data gathering and sharing. For the inquiry on the ethical underpinnings of this case, I develop a framework for domestic assistive robots for competent older adults drawn on the ethics of care. I assess that this type of robots could be ethically appraised attending to their impact on the well‐being and autonomy of users. I approach autonomy from a relational perspective, and I delve into the relationship between autonomy and well‐being through the concept of paternalism. I argue that this type of assistive robots should never act paternalistically. Given ROB‐IN great implications regarding privacy, I subsequently explore the ways in which the privacy of users should be respected in their interaction with assistive robots, focusing on the relation with autonomy and well‐being. Lastly, I highlight the need for avoiding ageism. This investigation focuses on aged users, but it is suggested that the situation of caregivers should be also the object of further investigations.

## 1 INTRODUCTION

Assistive robots are those that are intended to operate in care practices.^1^ Given the impact they are likely to have on care environments, and the moral relevance of care, it is not surprising that ethical inquiry on assistive robotics have significantly gained momentum in last years.^2^ Among other traditions, care ethics have been one of the most influential frameworks for this analysis.^3^ Assistive robots can be classified attending to its context of use, its functions, and its intended users,^4^ and those aspects are arguably relevant for their ethical appraisal.

The ROB‐IN project is a Spanish, public‐funded research project^5^ devoted to the design of assistive robots capable of accompanying and helping aged people in their own homes. The expected users are recipients of public home assistance who may use extra help for their daily tasks and their routine health monitoring. The general preliminary applications of the introduction of the robot are: “(1) physical help, like bringing items, help with feeding and drinking; (2) engagement in social interactions, like making calls to relatives, showing family photos, etc. (3) encouragement of physical and cognitive training, by using the robot to administer and evaluate particular exercises; and lastly, (4) information gathering to help the caregiver.”^6^ Regarding this last function, the robot will be capable of collecting relevant information from the daily life of the user, enhancing the fluidity of the information‐sharing with healthcare staff or family members. It could also improve the quality of the information, since it may track some events that can go unnoticed by the user herself. It may be useful for the understanding of the person's own health status.

While ROB‐IN seems to be a valuable ally for improving the quality of life and independence for its users, its features also raise important ethical issues that need to be addressed. These issues include autonomy fostering, privacy concerns, and ageism risks; concerns that are commonplace in care roboethics. As such, I propose an ethical analysis suitable for home assistive robots for competent older people following a care ethics and relational autonomy framework. ROB‐IN case is proposed as a useful example to illustrate the advantages of this framework.

I define a concept of good care that delves into the dyadic between autonomy and well‐being, and I argue why these two intertwined concepts may be adequate tools for the ethical analysis of ROB‐IN and other equivalent robots. Furthermore, I assess the relation between well‐being and autonomy through the idea of paternalism. Subsequently, I consider the issue of privacy in relation with autonomy and well‐being. In addition, I deal with the risk of ageism, considering the different ways it may create ethically troublesome situations in relation to paternalism, autonomy, and success of care endeavors. To conclude, I put forward some indicative recommendations for the ethical designing and implementation of home assistive robots for nondependent elder users.

## 2 CARE ETHICS SITUATED: A CONCEPT OF GOOD CARE FOR ASSISTIVE ROBOTS

According to the canonical definition by Berenice Fisher and Joan Tronto, care is “a species activity that includes everything that we do to maintain, continue, and repair our ‘world’ so that we can live in it as well as possible.”^7^ Here, I highlight three main aspects of good care, rooted in ethics of care tradition. First, care is linked to the good life (“to live as well as possible”). Thus, well‐being can be understood as a key indicator for addressing the success of the introduction of assistive robots in the home care context. Second, care is a collective endeavor (“everything *we* do”), so the situation of everyone implied is intertwined and affects one another. Third, good care should not imply oppression or undue paternalism. Autonomy in care ethics entails a reconfiguration of the idea of individual agent towards a socially embedded understanding of the self.^8^

Autonomy is a complex and very disputed term in ethics literature. Autonomy has been traditionally understood as the possibility for self‐governance, including the possibility and capability of understanding relevant information, elaborating a critical and personal reflection on it, and taking self‐governed decisions.^9^ In this vein, for autonomy to be possible, one capital element is the absence of unwarranted interference. Lack of information, manipulation, coercion, undue paternalism, and other elements may hinder autonomy, and people can (voluntarily or not) impede the autonomy of others by these means.

Autonomy does not equate freedom. While they may overlap (e.g., regarding the relevance of noninterference), they do not need to happen simultaneously.^10^ Freedom can be understood both in its negative sense (liberty from external interference) and in its positive sense (the existence of a political space for mastering one's self‐chosen life project).^11^ While it is reasonable to wonder if a robot can improve or hinder both types of freedom,^12^ here I am more concerned with the impact on autonomy. Warranting that a robot will not illegitimately interfere with user's decisions may be one way to uphold freedom, but it does not guarantee that autonomy is being respected and promoted. This is especially true if we follow a relational approach to autonomy.

If understood relationally, autonomy is not a condition of an isolated individual, but always embedded in a social web that constitutes the self. Relational approaches to autonomy are diverse, but I will mainly draw on two ideas, which are widely shared by all accounts; namely, that autonomy can be assessed through the attention to some conditions that shape its development^13^ and that those conditions are deeply dependent on the context and relations in which the person is situated.^14^ These capacities can be developed or hampered by the social surroundings of the individual. I suggest focusing on how an assistive robot such as ROB‐IN would affect these capacities in a way that autonomy overall is respected and, ideally, widened.

Well‐being is not an obvious term in ethics as well.^15^ This is a useful concept in the context of assistive robotics, since they are at least partly devoted to improve the quality of life of users. Well‐being is a broad idea that can encompass this general endeavor, but it needs further clarification to articulate it as a well‐grounded ethical guidance. First, well‐being can be understood as a subjective phenomenon, that is, a good that depends more or less irreducibly on inclinations, decisions, or preferences of the person.^16^ However, this notion of well‐being does not pair well with the relational version of autonomy. Respecting the self‐determined interests of the person as the last resort for determining well‐being conditions does not guarantee a proper promotion of autonomy, when understood in a relational sense.

For this reason, a more objective notion of well‐being is preferred. As argued by Aimee Van Wynsberghe,^17^ ethics of care is capable of rationally determining some values that do not depend on personal preferences. While Human–Robot Interaction studies on assistive robotics tend to give significant ethical relevance to users' preferences, Van Wynsberghe considers that they should be weighted with the objective, rationally justified values of care—that is, we can state some objective account of good care. In the context of assistive robots, the scope to which they can contribute to well‐being is arguably limited. They can make some activities of daily life smoother and more pleasant, while also improving some health‐related tasks. For the purpose of this article, then, I define well‐being as the expanding of the possibilities for pleasure and health. While the content of both objectives (particularly pleasure) still depends on the particular individual, the general inclination is understood as an objectively justified criterion for assistive robots.

I assume, then, that one of the objectives of the assistive robot is to widen the well‐being of the user, understood as the expanding of the possibilities for pleasure and health, because these are two markers of well‐being arguably attainable by the robot. On the one hand, the robot may contribute to making some hours per day more comfortable, friendly, and fun for the user; on the other hand, the robot is intended to collaborate in making the healthcare relationships more fluid and successful, thus contributing to the good health overall. Most relevantly, I understand well‐being to be fundamentally linked with autonomy, as it will be explained in the next section.

## 3 BALANCE BETWEEN AUTONOMY AND WELL‐BEING: HOW TO HANDLE PATERNALISM

Following the classical definition by Gerald Dworkin, paternalism is “the interference with a person's liberty of actions justified by reasons referring exclusively to the welfare, good, happiness, needs, interests or values of the person being coerced.”^18^ Given that, paternalism involves two indispensable features: to interfere in someone else's freedom or autonomy and to be motivated by the alleged well‐being of this person. Paternalism is not always morally negligent. An example of legitimate, even desirable, paternalism might be the parents forcing their child to wear a helmet when riding a bike, even when the child refuses to do it. Paternalism can even happen in the form of an interference in freedom that improves future conditions of autonomy (e.g., this same parent makes efforts to promote their child's reading skills even if they seem to dislike books, knowing that literacy will improve their capacity to act autonomously in life). Paternalism seems generally adequate when it implies a competent, responsible person taking care of an incompetent, vulnerable person on behalf of their best interests. However, there are cases in which the legitimacy of paternalism is tricky and contestable.

In ROB‐IN's case, as it happens in other assistive robots' intended scenarios of use, users are not under any circumstances that could call for an overriding of the presumption of their capacity for autonomy, such as very young age. Consequently, they can be assumed to have the legitimacy to decide over their own well‐being in general terms. Sometimes the robot may be useful for helping them to care for their own well‐being, such as reminding them to take their medicines or detecting some health‐related markers that may go unnoticed by the person herself. While these are desirable actions in general, in ROB‐IN and similar cases, I will argue that the search for the user's well‐being needs to be always subordinated to autonomy.

First, as stated, users should be presumed to be capable of autonomous behavior.^19^ Even if there can be cases in which the person is not fully aware of her situation or does not take the seemingly better choices, she does not comply with any relevant criteria for denying the presumption of autonomy. Older people may be subjected to prejudice regarding their capacity for autonomy (see Section 5), thus, the risk of undue paternalism in healthcare interventions is notable. In case the user is no longer a capable patient, they will no longer be a suitable user for this type of robot.

Second, even in situations in which human paternalism is justified, there are convincing reasons for defending that machine paternalism is not equal to human paternalism. Some interventions on freedom can actually improve the capacities for autonomy, and the evaluation on the ethical convenience of this type of interventions are common in human‐to‐human relationships, and also in decisions related to healthcare. However, there are reasons to believe that human‐to‐robot relationships may be subjected to a more restrictive ethical criteria in this regard. Certainly, the robot may be able to “know” better than the human what are their best interests in some situations. For example, the robot may know for sure that is convenient for the user to avoid adding salt to her meals, while the user can either forget about it, disregard its importance due to psychological biases (“I'm not that sick, I feel good”), or simply decide not to take care of herself in this way. Admittedly, it is not odd to imagine a situation in which another person (say, a relative) tries to make the user remove the salt, by different means, and the legitimacy of this act is not off the table. Would this situation be the same if the paternalistic action is performed by a robot such as ROB‐IN?

Recently, Tsung‐Hsing Ho^20^ has argued that there is a relevant difference between the moral significance of a paternalistic act done by a robot and an equivalent one done by a human. The main reason is that, for paternalism to be morally satisfactory, there are agent‐relative attributes that should be met. For example, while a family member seems to be authorized to forbid a child to eat too many candies, it is less clear that a stranger has the legitimacy to order this same child in the street to stop eating candy “for his own good.” Ho states that for a paternalistic act to be permissible, an interpersonal relation of a certain kind needs to be in place. Of course, not all relationships comply with the relevant criteria of legitimacy, and hence, some human paternalistic acts are also blameworthy. The key idea is that, for robots like ROB‐IN, there is no possibility for this interpersonal relation.^21^

Third, I do not consider that the introduction of the robot would render unnecessary the participation of other people in the process. The user would probably be embedded in general networks of social connectedness and care, such as health institutions or family relationships. The robot must not imply a marginalization from any of these sources of socialization. In this sense, there is no need to delegate the complicated conflicts between well‐being and autonomy that may arise to the robot. They should always be managed by the appropriate people.

Finally, it is important to note that a relational account of autonomy, such as the one I am proposing here, blurs somehow the clear opposition between autonomy and well‐being. On the one hand, I assume that the possibility of developing an autonomous way of managing his own life is a relevant part of overall wellness; consequently, paternalism in itself can turn out problematic. On the other hand, not all interferences in the user's will or freedom do override her autonomy. If autonomy is constructed in a context of truthful and trustworthy relationships, the collaboration between different agents is part of the path to encounter an autonomous decision.

In conclusion, ROB‐IN and other similar robots should not be allowed to act in a paternalistic way in the absence of human supervision. Nevertheless, as highlighted before, a relational autonomy framework helps to situate any robotic action in the context of the general social network in which the person is inserted. In this sense, while the robot should not be programmed to overcome user's decision in any sense, the door remains open for the robot to participate in human potentially paternalistic actions subjected to their own criteria for ethical acceptance.

To articulate this general guideline, the robot's capacities should be subjected to further inquiry. ROB‐IN is a useful example of two particularities that are shared by a considerable number of contemporary assistive robotics: it has a significant capacity for data gathering and sharing, and it is intended for older nondependent, cognitively competent adults. For this reason, in what follows, I propose an account on privacy that is built upon the relational autonomy and paternalism framework explained above. Subsequently, I justify why there is a considerable risk of ageism in robot design for elder people and I propose some recommendations for avoiding it.

## 4 PRIVACY AND AUTONOMY: A TWO‐WAY PATH

ROB‐IN presents considerable potential ethical problems regarding privacy, for it is supposed to gather information about health status, it should operate on the private homes of the users, and it could be programmed to share information with other people, for example, a physician. In the section that follows, I will examine privacy not as a unique, absolute value, but in relation to autonomy and well‐being. After this analysis, it will be possible to propose some preliminary recommendations for the design and implementation of assistive robots for older people.

Privacy has been one of the typical ethical concerns since the first reflections on the possibilities of using robots for the care of older people.^22^ While privacy is a major worry in the ethics of disruptive technologies, and it is widely present in all recommendations and regulations, its meaning is not as straightforward as it may seem. Literature around privacy has made several distinctions on possible meanings of the term itself. For example, Rueben et al.^23^ distinguished between informational, physical, psychological, and social privacy for a taxonomy of privacy‐sensitive robotics. Ella Kolkowska and Miranda Kajtazi^24^ proposed to evaluate privacy in smart homes for the aged distinguishing privacy of the person or bodily privacy, privacy of personal behavior in sensitive matters, privacy of personal communication, and privacy of personal data. Elaine Sedenberg and colleagues^25^ differentiated between physical, decisional, psychological/mental, and informational privacy for therapeutic home robots' ethical evaluation. Christoph Lutz and Aurelia Tamó‐Larrieux^26^ have analyzed users' preferences for privacy using only two useful concepts of privacy: physical and informational.

Here, I distinguish between *intimate privacy* and *data protection*. These two aspects are not necessarily covering all nuances of privacy, but they are arguably the most important when it comes to the types of robots under consideration in this article. In these robots, neither psychological and mental state indicators nor biometrical markers will be collected. The example case, ROB‐IN, focuses on the gathering of health‐related data from behavior and routines. Because of the remarkably sensitive character of these gatherings, as I will explain further on, privacy standards are quite restrictive.

*Intimate privacy* refers to the possibility of being left alone when one wants to be so. Intimate privacy is needed to develop and exercise some abilities related to autonomy, such as self‐reflection, rational evaluation of information, feeling of safety and independence, and the absence of invasive coercion of another person. Intimacy has also been considered to play a central role in the development of self‐awareness and social skills.^27^

There is consolidated agreement on the literature about the human tendency to anthropomorphize robots.^28^ This bias is one of the reasons that leads us to expect that the presence of a robot may impact substantially the intimate privacy of users. ROB‐IN, as other assistive robots, is to be inserted in the private home of users. One's own home is an extremely delicate space regarding this intimate privacy. People may feel invaded, uneasy, or surveilled by the presence of the robot in their very personal space. Aged users may feel uncomfortable if they are being monitored either by a camera or a robot, and they can change their comportment for this reason.^29^

The robot should behave in a way that acknowledges this fact. Daseul Yang and colleagues^30^ have found that there are, at least, three components of a robot's behavior that clearly impact people's sense of privacy and trust in it: gaze (people do not like to feel stared at by others, even less by robots), distance (the robot should respect cultural norms regarding bodily personal space), and clarity in expressing intent. This last feature is relevant since it has also been found that sometimes people are not aware that they are being recorded or filmed by the robot if they are not warned about it beforehand. Among other recommendations, they propose that a robot should always turn back when someone is naked or when it detects signs associated with private conversations. If the presence of a robot may be of disturbance in some cases, probably to have control over it could be a remedy. Some research has shown that control over the robot is a main indicator of users' willingness to make use of it.^31^ The user should always be capable of indicating to the robot when and where it should go away or stop any action.

The acknowledgment of the risk of disturbing intimate privacy is twofold. On the one hand, invading one's intimate privacy is a harm in itself. It affects the self‐conception of the person. It can make them feel threatened or insecure in their own safe space, their homes. It can provoke stress and discomfort and hinder some of the abilities for autonomy. On the other hand, invading intimate privacy may also provoke rejection of the robot. People tend to decrease their trust in a robot if they feel that it can be invasive or even dangerous to their intimate self.^32^ So, the reason for searching for a noninvasive robot in the homeplace is both ethically and pragmatically founded.

*Data protection* refers to the correct management of personal data gathered by the robot. According to European General Data Protection Regulation (GDPR),^33^ personal data are “any information relating to an identified or identifiable natural person (data subject).” Besides compliance with the GDPR (as any robot affected by European legislation), data protection should be aligned with the general objective of enhancing the autonomy and well‐being of the user.

Following a relational autonomy framework, autonomy is not only assessed through concrete actions free from undue interference (e.g., the user signs an informed consent for the treatment of their data), but it should be also inquired whether the relationships enabled by the robot contribute to the development of abilities and capacities related to autonomy. In this sense, considering both intimate privacy and data protection, I propose to analyze the following features for any assistive robot that is intended to perform in private home of aged users:

*Self‐awareness*: for autonomy, people need to have considerable and reasonable knowledge of their own patterns of behavior, their own feelings and thoughts, and their weaknesses and strengths. Likewise, for being autonomous in managing their own health, people should have access to the best information regarding their health state. This information should be (1) understandable, thus adapted to the cognitive abilities of the user, (2) relevant to the health care, and (3) desired, meaning that the user should only know what they want to know, and they hold a right not to know.^34^ For achieving these objectives, the user does not need to be alone in the process. On the contrary, the collaboration among the people involved is essential for them to correctly understand their health state and thus be capable of taking autonomous decisions about it. Accordingly, robots like ROB‐IN may significantly contribute to this self‐awareness, as long as the information is shared with the relevant people, the patient trusts the people whom the information is shared with, the information is always strictly relevant for their health care, and the patient is always actively participating in the process.

Self‐awareness is closely related with *deception*. Deception may happen if the user thinks that the robot can do something that it is not actually capable of doing. There is no absolute consensus among scholars regarding if deception will necessarily happen in robotics care, or if, when it happens, it will always be harmful.^35^ When understood in relation to autonomy, deception is arguably undesirable. Deception may be the source of uninformed or ill‐informed decisions and a sense of betrayal or lack of trust in the robot after a failure. If there is deception, autonomy is compromised in several ways. The information available to the person is biased. The trust in the robot is not grounded in objective capacities but in a certain amount of fiction. The self‐concept of the person may be harmed if they discover they have been “fooled” by a machine. Henceforth, this harm should be prevented. It is necessary that the robot is able to express its actual capacities, and clearly explain to the person if they are asking for something it is not capable of doing. While bonding with objects and anthropomorphizing are phenomena widely documented and probably not fully avoidable, it is undesirable for the person to have the mistaken conception that the robot has emotions or that it is capable of developing a relationship of mutual affection.

*Self‐confidence and self‐esteem*: for autonomy, people need to trust their own capacity for understanding their situation and taking reasonably accurate decisions about it.^36^ Note that self‐esteem is not totally equivalent to the actual capacity of understanding and deciding. A person may be capable of understanding their situation and sensibly deciding what is best for her but lack the confidence to follow their own thoughts and intuitions. Self‐esteem is sustained by other's people trust in the person; if no one thinks I can manage my own life, it is less likely that I trust my own criteria. A robot such as ROB‐IN may be an ally in sustaining and enhancing the self‐esteem of the user. This can be done by encouraging and empowering communication, inciting independent decision‐making, and avoiding interfering in their decisions. Particularly, a high level of the user's control over the flux of information among healthcare providers and relatives is recommendable.

*Social support*: A relational account of autonomy understands the subject always embedded in a network of relations that shape the abilities for autonomy. People need to be able to ask for help when needed and to belong to a community that treasures their autonomy. A robot like ROB‐IN may be a help in this regard, facilitating the fluidity and speed of communication and enabling contact with other people through telephone, video, or messaging.

*Trust and acceptable delegation*: most people cannot have all the expert knowledge about everything that affects their lives. Sometimes this knowledge is too broad and requires the involvement of several experts or it simply exceeds the cognitive capacities of the person. This is normally the case of medical knowledge: people who are not medical experts do not possess the specialized knowledge about their health condition. However, this specific knowledge is not necessary when communicating with their healthcare providers. What they do need is to have sufficient trust in the health institutional system and its staff so that they can believe that the information provided is truthful and beneficial.

This is also the case of technological knowledge. Transparency is not enough to guarantee the protection of the user if the user lacks the complicated technical knowledge that lies behind most technological tools. Consequently, there is a need for a solid and trustworthy system that provides support to users.^37^ Sometimes, expert knowledge will be needed for reaching an adequate decision. No expert knowledge can surpass the control of the user on their own decisions; on the contrary, expert knowledge is expected to maximize the user's capacity to accomplish an autonomous path of action. ROB‐IN will not take decisions based on expert health information. What it will do is to improve the fluidity of the transmission of relevant information from the user to their physicians and to enhance the user's capacity to access reliable health information from different sources by avoiding the non‐reliable ones, for example, filtering online resources.

In what follows, I propose a specific account on the particularities of the intended users' situation. Age may be the source of mistreatment if ageist prejudices are present in the design and implementation of robots. Since aged users are one of the most prominent target users of contemporary assistive robotics development, these considerations are significantly relevant in the field.

## 5 VULNERABILITY AND AGEISM: AVOIDING MASKED PATERNALISM

Aged people are typically considered especially vulnerable because they are likely to be affected by factors such as declining cognitive abilities, lack of familiarization with new technologies, or marginalization from relevant social networks.^38^ It is worth noting that all vulnerable situations are conditioned by social factors. Even medical conditions are not purely biological processes: they are affected by the social determinants of health.^39^ Successful assistive robotics should not widen the existing vulnerabilities but contribute to take care of the existing ones, aiming to reduce them.

While it seems true that age usually comes with certain specific vulnerabilities, for example, physical fragility, it is not clear whether labeling older people as homogenously “vulnerable” is always in their best interest.^40^ Prejudices may cause mistreatment of aged patients, based on inaccurate ideas of what is it to be aged and even failing to respect their autonomy.^41^ An adequate account of vulnerability is needed for a correct understanding of the elderly and their needs.

Ethical literature about vulnerability stressed the problems that may arise from an acritical treatment of vulnerability,^42^ including stigmatization, revictimization, and disempowerment. In the case of aged people, these problems are entangled with ageism. Ageism may be defined as the “stereotypes (how we think), prejudice (how we feel) and discrimination (how we act) directed towards people on the basis of their age.”^43^ Ageism may take the form of active contempt (“aged people are incapable,” “aged people are a burden”), but this is not the only possibility. Here, I am more interested in the ageism that may result from good intentions, even the unnoticed ones, because this is the type of ageism that can surreptitiously arise in projects that seek out the well‐being of aged people. I will refer to this type of ageism as *masked ageism*. This may take the following manifestations:

*Undue generalization*: literature about vulnerability has pointed out the problem of homogenizing a group based on solely one shared characteristic.^44^ This focuses on only one particular feature that may cause the dismissal of other potentially relevant traits. In the case of aged people, generalizing their needs and desires because of their age could lead to overlooking the importance of other relevant personal qualities. For example, it would be illegitimate to presume a universal desire to receive help for certain daily tasks such as cooking, regardless of the person's habits, capacities, and preferences. In assistive robotics, this may be overcome by the personalization of the robot and the collaboration with the user and the people close to her in defining the tasks and roles that the robot should have in the household. It should be noted that there is a trade‐off between data protection and personalization^45^ that should be balanced by taking into account the user's preferences and situation.

*Disempowerment*: Paternalistic interventions may reduce the capacity of people being in control of their own lives, even causing learned helplessness.^46^ Masked ageism may contribute to this by assuming a standard need for paternalistic support for all aged people and reducing their possibilities for autonomy and self‐governing. Catriona Mackenzie, Wendy Rogers, and Susan Dodds speak about “pathogenic vulnerability,” which happens when a flawed intervention in a vulnerable situation results in a reinforcement of existing vulnerabilities or the creation of new ones.^47^ It is relevant to ensure that an assistive robot will not contribute to a decrease in certain autonomy abilities, as developed in the previous sections.

*Marginalization*: ageism, like other forms of discrimination, may appear as the separation of a group of people from meaningful social networks. In the case of masked ageism, some interventions initially aimed to tackle the specific needs of aged people may cause their separation from the rest of the population and therefore make it difficult for their participation in society and the development of autonomous abilities. An assistive robot, being social or not, must not reduce the social engagement and participation in relevant social networks of the user. A desirable outcome will be to enhance the capacity to communicate and be in touch with people through telephone or other means.

Successful aging model^48^ can be a source of inspiration for a more respectful account of older age in assistive robotics. This model highlights the relevance of nonbiological factors in determining the way in which people age. First, it helps to avoid a monolithic, potentially reductionist view on how age impacts the life of individuals, shifting the focus to the elements that shape the quality of life besides biological aging. Second, it brings to the forefront of health care the relevance of active lifestyle and belonging to significant social networks, among other aspects. In this sense, the objectives of assistive robots can be framed as a form of help to maintain or develop those healthy parameters, rather than assuming a common need of direct assistance for every elder user.

## 6 CONCLUSIONS

ROB‐IN is a robot intended to be used by non‐dependent users in their own homes. For an ethically informed understanding of the benefits and risks of ROB‐IN, I have proposed to focus on the possibilities of home assistive robots for older users to enhance autonomy and well‐being, following a care ethics' evaluation framework. Autonomy is understood from a relational perspective, and the user's well‐being and autonomy are intertwined. Paternalism directly performed by a robot, in absence of significant human participation, has been stated as illegitimate in any scenario implying competent older adults.

Privacy has been analyzed with priority, because great part of the benefits that may arise from the implementation of ROB‐IN are related to health data gathering and management, and because privacy is one of the main issues in the literature on assistive robotics. For the purposes of this case, two concepts of privacy have been presented: intimate privacy and data management. Both have been scrutinized according to their impact on relevant autonomy‐related abilities that are likely to be affected. Complementary, ageism has been diagnosed as a relevant risk that can lead to undue paternalism over aged users and that may even take the form of masked ageism hiding behind good intentions towards the care of elder users.

Following these recommendations, the ROB‐IN project will significantly increase its ethical adequacy. Since this is a prospective analysis, further research will be needed to longitudinally study ROB‐IN's implications. Similarly, analogous assessment should be performed regarding the impact ROB‐IN may have on formal and informal caregivers.

Assistive robots may contribute to improve both the autonomy and well‐being of users, but ethical scrutiny will be needed throughout to avoid undesirable counter effects. This article aims to contribute to this endeavor. While this is a situated analysis that responds to the particularities of ROB‐IN's and similar cases, I hope that it can be of significant interest for scholars on the ethics of assistive robotics.

## Footnotes

^1^
Pareto Boada, J., Román Maestre, B., & Torras Genís, C. (2021). The ethical issues of social assistive robotics: A critical literature review. *Technology in Society*, 67, 101726. https://doi.org/10.1016/j.techsoc.2021.101726

^2^
Vandemeulebroucke, T., Dierckx de Casterlé, B., & Gastmans, C. (2018). The use of care robots in aged care: A systematic review of argument‐based ethics literature*. Archives of Gerontology and Geriatrics*, *74*, 15–25. https://doi.org/10.1016/j.archger.2017.08.014; Pareto Boada, J, et al., op. cit. note 1.

^3^
Vallor, S. (2011). Carebots and caregivers: Sustaining the ethical ideal of care in the twenty‐first century. *Philosophy & Technology*, *24*, 251. https://doi.org/10.1007/s13347-011-0015-x; Van Wynsberghe, A. (2015). *Healthcare robots. Ethics, design and implementation*. Routledge.

^4^
Van Wynsberghe, op. cit. note 3.

^5^
For more information about the project, see ROB‐IN Project (https://projecte-robin.github.io/).

^6^
IRI—ROB‐IN: Robots para la asistencia continua y personalizada capaces de explicarse a sí mismos (csic.es).

^7^
Fisher, B., & Tronto, J. (1990). Towards a feminist theory of caring. In Abel, E. K. and Nelson, M. K. (Eds), *Circles of care: Work and identity in women's lives*. State University of New York Press. Cited in Tronto, J. (2013). *Caring Democracy: Markets, Equality, and Justice*. New York University Press, p. 19.

^8^
Keller, J. (1997). Autonomy, relationality, and feminist ethics. *Hypatia*, *12*(2), 152–164. https://doi.org/10.1111/j.1527-2001.1997.tb00024.x; In the present analysis, the focus is placed on aged users. The caregiver's situation will be assessed in further investigations.

^9^
Beauchamp, T., & Childress, J. (2019), *Principles of biomedical ethics* (8th ed.). Oxford University Press.

^10^
Véliz, C., Maslen, H., Essman, M., Smith Taillie, L., & Savulescu, J. (2019). Sugar, taxes, and choice. *Hastings Center Report*, *49*(6), 22–31. https://doi.org/10.1002/hast.1067

^11^
Berlin, I. (1969). Two concepts of liberty. In *Four essays on liberty* (pp. 121–154). Oxford University Press.

^12^
Pareto, J., & Coeckelbergh, M. (2024). Social assistive robotics: an ethical and political inquiry through the lens of freedom. *International Journal of Social Robotics*. https://doi.org/10.1007/s12369-024-01161-x

^13^
Nedelsky, J. (1989). Reconceiving autonomy: Sources, thoughts and possibilities. *Yale Journal of Law & Feminism*, *1*, 7–36.

^14^
Mackenzie, C., & Stoljar, N. (2000), *Relational autonomy. Feminist perspectives on autonomy, agency, and the social self*. Oxford University Press; Stoljar, N. (2022). Feminist perspectives on autonomy. In E. N. Zalta & U. Nodelman (Eds.), *The stanford Encyclopedia of philosophy* (Winter 2022 Edition). https://plato.stanford.edu/archives/win2022/entries/feminism-autonomy/.

^15^
Crisp, R., (2021). Well‐being. In E. N. Zalta (ed.), *The stanford Encyclopedia of philosophy* (Winter 2021 ed.). The Stanford Encyclopedia of Philosophy. https://plato.stanford.edu/archives/win2021/entries/well-being/.

^16^
Ibid.

^17^
Van Wynsberghe, op. cit. note 3.

^18^
Dworkin, G. (1972). Paternalism. *The Monist*, *56*(1), 64–84, p. 65.

^19^
Note that there are robots who are intended to help people with different types of cognitive diversity, ranging for autism spectrum conditions to cognitive decline. Robots for dementia patients are a particularly relevant field, and they can easily overlap with robots for the elder. The framework developed here is not suitable for any adult who is diagnosed with significant cognitive decline, since it is based in the presumption of the absence of any clear ground that would justify a higher intervention on someone's freedom for encouraging her autonomy.

^20^
Ho, T. H. (2022). Moral difference between humans and robots: Paternalism and human‐relative reason. *AI & Society*, *37*(4), 1533–1543. https://doi.org/10.1007/s00146-021-01231-y

^21^
Ibid: 1540; Paternalistic relationships can also happen between a person and another type of entity, such as institutions and states, which are typically the object of discussion in paternalism literature. Ho's arguments can be complemented by pointing out that robots also lack the capacity to engage in the type of relationships that give rise to legitimacy in institutional paternalism, such as democratic policy‐making, answerability, or promotion of the common good.

^22^
Heuer, T., Schiering, I., & Gerndt, R. (2018). Privacy and socially assistive robots—A meta study. In M. Hansen, E. Kosta, I. Nai‐Fovino, S. Fischer‐Hübner (Eds.), *Privacy and identity management. the smart revolution. privacy and identity*. Springer. https://doi.org/10.1007/978-3-319-92925-5_18

^23^
Rueben, M., Grimm, C., Bernieri, F. J., & Smart, W. D. (2017). A taxonomy of privacy constructs for privacy‐sensitive robotics*. ArXiv*, abs/1701.00841. https://doi.org/10.48550/arXiv.1701.00841

^24^
Kolkowska, E., and Kajtazi, M. (2015). Privacy dimensions in design of smart home systems for elderly people. *WISP 2015 Proceedings*. 17. https://aisel.aisnet.org/wisp2015/17

^25^
Sedenberg, E., Chuang, J., & Mulligan, D. (2016). Designing commercial therapeutic robots for privacy preserving systems and ethical research practices within the home. *International Journal of Social Robotics*, *8*, 575–587. https://doi.org/10.1007/s12369-016-0362-y

^26^
Lutz, C., & Tamó‐Larrieux, A. (2020). The robot privacy paradox: Understanding how privacy concerns shape intentions to use social robots. *Human‐Machine Communication*, *1*, 87–111. https://doi.org/10.30658/hmc.1.6

^27^
Conill, J. (2016). La intimidad corporal y sus bases neurobiológicas. *Pensamiento. Revista de Investigación e Información Filosófica*, *72* (273 Extra), 789–807. https://doi.org/10.14422/pen.v72.i273.y2016.002

^28^
Wester, J., Schneiders, E., & van Berkel, N. (2023). Perceived moral agency of non‐moral entities: Implications and future research directions for social robots. In *HRI ’23: ACM/IEEE International Conference on Human‐Robot Interaction (HRI): Workshop on Perspectives on Moral Agency in Human‐Robot Interaction* (pp. 1–3).

^29^
Caine, K., Šabanovic, S., & Carter, M. (2012). The effect of monitoring by cameras and robots on the privacy enhancing behaviors of older adults. In *Proceedings of the seventh annual ACM/IEEE International Conference on Human‐Robot Interaction* (pp. 343–350). https://doi.org/10.1145/2157689.2157807

^30^
Yang, D., Chae, Y. J., Kim, D., Lim, Y., Kim, D. H., Kim, C., Park, S‐K., & Nam, C. (2022). Effects of social behaviors of robots in privacy‐sensitive situations. *International Journal of Social Robotics*, *14*, 589–602. https://doi.org/10.1007/s12369-021-00809-2

^31^
Deutsch, I., Erel, H., Paz, M., Hoffman, G., & Zuckerman, O. (2019). Home robotic devices for older adults: Opportunities and concerns. *Computers in Human Behavior*, *98*, 122–133. https://doi.org/10.1016/j.chb.2019.04.002; Coghlan, S., Waycott, J., Lazar, A., & Barbosa Neves, B. (2021). Dignity, autonomy, and style of company: Dimensions older adults consider for robot companions. *Proceedings of the ACM on Human‐Computer Interaction*, *5*(CSCW1), 1–25. https://doi.org/10.1145/3449178

^32^
Etemad‐Sajadi, R., Soussan, A., & Schöpfer, T. (2022). How ethical issues raised by human–robot interaction can impact the intention to use the robot? *International Journal of Social Robotics*, *14*(4), 1103–1115. https://doi.org/10.1007/s12369-021-00857-8

^33^
European Parliament and European Council. (2016). *Regulation (Eu) 2016/679 of the European Parliament and of the Council of 27 April 2016 on the protection of natural persons with regard to the processing of personal data and on the free movement of such data, and repealing Directive 95/46/EC (General Data Protection Regulation)*. EUR‐Lex–02016R0679‐20160504—EN—EUR‐Lex (europa.eu).

^34^
Andorno, R. (2004). The right not to know: An autonomy based approach. *Journal of Medical Ethics*, *30*(5), 435–439. https://doi.org/10.1136/jme.2002.001578

^35^
Coeckelbergh, M. (2012). Are emotional robots deceptive? *IEEE Transactions on Affective Computing*, *3*, 388–393. https://doi.org/10.1109/T-AFFC.2011.29; Vandemeulebroucke, T., et al., op. cit. note 2; Segers, S. (2022). Robot technology for the elderly and the value of veracity: Disruptive technology or reinvigorating entrenched principles? *Science and Engineering Ethics*, *28*(6), 64. https://doi.org/10.1007/s11948-022-00420-2

^36^
McLeod, C., & Sherwin, S. (2000). Relational autonomy, self‐trust, and health care for patients who are oppressed. In C. Mackenzie & N. Stoljar (Eds.), *Relational autonomy: Feminist perspectives on autonomy, agency, and the social self* (pp. 259–279). Cambridge University Press.

^37^
Degli Esposti, S., & Arroyo, D. (2022). Trustworthy humans and machines. Vulnerable trustors and the need for trustee competence, integrity, and benevolence in digital systems. In L. Viola & P. Laidler (Eds.), *Trust and transparency in an age of surveillance* (pp. 201–220). Taylor & Francis. https://doi.org/10.4324/9781003120827-15

^38^
Fernandes Barbosa, K. T., Rodrigues Lopes de Oliveira, F. M., Melo Fernandes, M. G. (2019). Vulnerability of the elderly: A conceptual analysis. *Revista Brasileira de Enfermagem*, *72*(Suppl 2), 337–344. https://doi.org/10.1590/0034-7167-2018-0728

^39^
Marmot, M. (2005). Social determinants of health inequalities. *The Lancet*, *365*(9464), 1099–1104. https://doi.org/10.1016/S0140-6736(05)71146-6

^40^
Fineman, M. A. (2012). Elderly as vulnerable: Rethinking the nature of individual and societal responsibility. *Elder Law Journal*, *20*, 71–142. https://doi.org/10.2139/ssrn.2088159

^41^
Agich, G. (2003). *Dependence and autonomy in old age: An ethical framework for long‐term care*. Cambridge University Press; Cooley, D. R. (2015). Elder abuse and vulnerability: Avoiding illicit paternalism in healthcare, medical research, and life. *Ethics, Medicine and Public Health*, *1*(1), 102–112. https://doi.org/10.1016/j.jemep.2014.09.003

^42^
Virokannas, E., Liuski, S., & Kuronen, M. (2020). The contested concept of vulnerability—A literature review: *European Journal of Social Work*, *23*(2), 327–339. https://doi.org/10.1080/13691457.2018.1508001

^43^
WHO. (2021). *Global report on ageism*. World Health Organization. Licence: CC BY‐NC‐SA 3.0 IGO, p. XV.

^44^
Brown, K. (2011). ‘Vulnerability’: Handle with care. *Ethics and Social Welfare*, *5*(3), 313–321. https://doi.org/10.1080/17496535.2011.597165; Luna, F. (2009). Elucidating the concept of vulnerability: Layers not labels. *IJFAB: International Journal of Feminist Approaches to Bioethics*, *2*(1), 121–139. https://doi.org/10.3138/ijfab.2.1.121

^45^
Ashman, H., Brailsford, T., Cristea, A. I., Sheng, Q. Z., Stewart, C., Toms, E. G., & Wade, V. (2014). The ethical and social implications of personalization technologies for e‐learning. *Information & Management*, *51*(6), 819–832. https://doi.org/10.1016/j.im.2014.04.003

^46^
Faulkner, M. (2001). Empowerment, disempowerment and the care of older people. *Nursing Older People*, *13*(5), 18–20.

^47^
Mackenzie, C., Rogers, W., & Dodds, S. (2014). Introduction: What is vulnerability and why does it matter for moral theory. In C. Mackenzie, W. Rogers & S. Dodds (Eds.) *Vulnerability: New essays in ethics and feminist philosophy* (pp. 1–29). Oxford University Press.

^48^
Rowe, J. W., & Kahn, R. L. (1987). Human aging: Usual and successful. *Science*, *237*(4811), 143–149; Rowe, J. W., & Kahn, R. L. (1997). Successful aging. *The Gerontologist*, *37*(4), 433–440.

